# Gut Microbiota Modulation, Anti-Diabetic and Anti-Inflammatory Properties of Polyphenol Extract from Mung Bean Seed Coat (*Vigna radiata* L.)

**DOI:** 10.3390/nu14112275

**Published:** 2022-05-28

**Authors:** Suvimol Charoensiddhi, Wasaporn Preteseille Chanput, Sudathip Sae-tan

**Affiliations:** Department of Food Science and Technology, Faculty of Agro-Industry, Kasetsart University, 50 Ngamwongwan Road, Chatuchak, Bangkok 10900, Thailand; suvimol.ch@ku.th (S.C.); fagiwpc@ku.ac.th (W.P.C.)

**Keywords:** anti-diabetic, anti-inflammatory, gut microbiota, mung bean, polyphenols

## Abstract

The present study investigated the gut health, anti-diabetic, and anti-inflammatory activities of mung bean seed coat extract (MSE). MSE was obtained by pressurized liquid extraction (PLE) using 50% ethanol as the extracting solvent. After 24 h of *in vitro* human fecal fermentation, MSE exhibited higher productions of total short-chain fatty acids (SCFA) than those of the control group (CON) and other polyphenol-rich substrates, including gallic acid (GA) and vitexin (VIT) (*p* > 0.05), but still lower than the fructo-oligosaccharide (FOS). In 16S-rRNA next-generation sequencing, MSE regulated the composition of gut microbiota by stimulating the growth of the beneficial bacteria *Enterococcus*, *Ruminococcus*, *Blautia*, and *Bacteroides* and decreasing the growth of the potential pathogenic bacteria *Escherichia*-*Shigella*. Similarly, qPCR showed increased numbers of *Bifidobacterium*, *Lactobacillus*, *Faecalibacterium prausnitzii*, and *Prevotella*, compared with those of CON (*p* < 0.05). MSE also reduced reactive oxygen species and increased glucose uptake in insulin-resistant HepG2 cells dose-dependently. The anti-inflammatory activity of MSE was observed in LPS-stimulated THP-1 monocytes with the reduction of TNFα, IL-1β, IL-6, and IL-8 genes. The data demonstrated the potential applications of MSE as a dietary supplement with gut health benefits and its ability to mitigate diabetes and inflammatory-related diseases.

## 1. Introduction

The occurrence of gut microbiota dysbiosis was reported to contribute to host vulnerability, progressing to a large spectrum of infectious and noncommunicable diseases [1]. The gut ecosystem is a key player in maintaining host health through supplying numerous nutrients and modulating energy balance and immune responses [2]. Additionally, polyphenols can improve intestinal microbiota homeostasis and have beneficial effects on host health by protecting against pathogen invasion and the risk of obesity, type 2 diabetes, inflammatory bowel disease, cancer, and cardiovascular, liver, and central nervous system disorders [3]. Polyphenols from red wine could regulate the gut microbiota profile of patients with metabolic syndrome by increasing the number of *Bifidobacterium*, *Lactobacillus*, and butyrate-producing bacteria, such as *Faecalibacterium prausnitzii* and *Roseburia*, in feces [4]. Ray and Mukherjee [5] described the fates of dietary polyphenols and the link with intestinal microbial ecology, biological activities, and human well-being and disease.

Mung bean (*Vigna radiata*) is a summer pulse crop with a short growth cycle. It is one of the most important edible legume crops, consumed by most households in Asia [6]. Mung bean is a rich source of protein, carbohydrates, vitamins, and minerals and is consumed directly or processed into valuable products such as glass noodles, confectionary, or mung bean protein [7]. One of the major mung bean industry byproducts is mung bean seed coat, which is usually discarded despite being rich in polyphenols and dietary fiber. To avoid the waste of this functional raw material and encourage its use, the health benefits of mung bean seed coat have been explored. The anti-inflammatory effects of mung bean seed coat water extract were shown in LPS-induced inflammation in RAW264.7 cells, acute liver injury mice [8], and 3T3-L1 adipocytes [9]. The antioxidative activity of mung bean seed coat ethanolic extract was also reported in both *in vivo* and *in vitro* studies [10,11,12]. Mung bean seed coat powder and bound polyphenols from mung bean seed coat also exerted an inhibitory effect against α-amylase and α-glucosidase and lowered glycemic markers in diabetic *db/db* and KK-Ay mice [11,12,13]. A recent study in high-fat diet-induced obese mice showed that mung bean seed coat polysaccharides reduced fasting blood glucose, fat accumulation, and serum lipid levels, modulating the gut microbiota, particularly *Akkermansia* [14]. Similar findings with prediabetic mice were reported by Hou, Zhao [15]. Xie, Song [16] found that polysaccharide extracts from mung bean seed coat could increase mouse colon length, the production of short-chain fatty acids (SCFA), and the richness of the gut microbiota by maintaining intestinal health. Although there are few reports on the effects of mung bean seed coat and its polysaccharides on gut microbiota, this is not in the case for polyphenol extracts.

Thus, we aimed to investigate the bioactivities of mung bean seed coat polyphenol extract obtained from pressurized liquid extraction (PLE) using 50% ethanol as an extracting solvent, in terms of gut microbiota modulation and anti-diabetic and anti-inflammatory activities. These results would be a foundation for further investigations of the health benefits of mung bean seed coat extract (MSE) *in vivo* and human studies.

## 2. Materials and Methods

### 2.1. Materials and Chemicals

Mung bean seed coat was obtained from Kittitat Co., Ltd. Dulbecco’s modified Eagle’s medium (DMEM) and Roswell Park Memorial Institute Medium 1640 (RPMI) were obtained from Hyclone (Logan, UT, USA). Fetal bovine serum (FBS), penicillin–streptomycin, nonessential amino acids, and insulin human recombinant zinc were obtained from Gibco (Paisley, UK), whereas 2-deoxy-(N-(7-Nitrobenz-2-oxa-1,3-diazol-4-yl)amino)-D-glucose (2-NBDG) was obtained from Invitrogen (Waltham, CA, USA) and 2′,7′-dichlorofluorescin diacetate (DCFH-DA), vitexin (VIT) and isovitexin were obtained from Sigma-Aldrich (St. Louis, MO, USA). Lipopolysaccharides (LPS; Escherichia coli O111:B4) were obtained from Sigma-Aldrich (St. Louis, MO, USA). All other chemical reagents used were of analytical grade. Deionized water was used for preparing all solutions. All chemicals used in the study of SCFA and gut microbiota were of analytical or chromatographical grade, from Merck (Darmstadt, Germany) and Sigma.

### 2.2. Extraction of Polyphenols from Mung Bean Seed Coat

Mung bean seed coat was extracted using PLE at a temperature of 160 °C, with a pressure of 1500 psi, and with 50% ethanol for 10 min [17]. Then, the MSE was centrifuged at 7000 rpm, at room temperature for 10 min. The supernatant was collected and evaporated to remove the ethanol at 60 °C. Afterward, the MSE was freeze-dried and kept in an aluminum foil bag at 4 °C for further analyses.

### 2.3. Chemical Determination of Extract Contents

#### 2.3.1. Determination of Total Phenolic Content (TPC)

The TPC in MSE was determined using the Folin–Ciocalteu method [18]. Briefly, 75 μL of distilled water, 25 μL of MSE, and 25 μL of Folin–Ciocalteu reagent were added to the 96-well plate. After the solutions had been mixed and equilibrated for 6 min, 100 μL of 75 g/L Na2CO3 were added to each well. Solutions were incubated for 90 min. After 60 s shaking, the absorbance was measured at 765 nm using a microplate reader (TECAN, Infinite M200 Pro, Männedorf, Switzerland). Gallic acid (GA) was used as a reference standard and the result was expressed as mg gallic acid equivalent (GAE)/g MSE (dry weight; d.w.).

#### 2.3.2. Proximate Analysis

Proximate analysis of MSE was determined using the methods from AOAC [19]. The protein content was determined using the Kjeldahl method. The moisture content was determined using the oven drying method. The loss of weight obtained after drying was used to calculate the moisture content. The ash content was determined using 1 g of samples placed in a crucible of known weight inside a Gallenkamp furnace at 550 °C for 6 h. Each crucible was cooled in a desiccator and weighed. The fat content was measured using SoxtecTM with petroleum ether solvent. The carbohydrate content was calculated using the method of differences.

### 2.4. Determination of Antioxidant Activity

The antioxidant activity of MSE was determined using the 2,2′-azinobis-(3-ethylbenzothiazoline-6-sulfonic) acid (ABTS) assay according to Indracanti, Sivakumar [20]. Briefly, 10 μL of MSE extract was mixed with 190 μL of ABTS solution. The mixed solutions were incubated at room temperature for 6 min in the dark. Then, the absorbance was measured at 734 nm using a microplate reader (TECAN, Infinite M200 Pro, Männedorf, Switzerland). Trolox was used as a reference standard, and the result was expressed as mg trolox equivalent (TE)/g MSE (d.w.).

### 2.5. Gut Microbiota Modulation

#### 2.5.1. *In Vitro* Human Gut Model

Fresh fecal samples were provided by three healthy human volunteers. The volunteers had no dietary restrictions and had not taken antibiotics or probiotics for at least 3threemonths before donating. Anaerobic conditions were maintained throughout the preparation of fermentation using an anaerobic chamber (Bactron IV Anaerobic Chamber Sheldon Manufacturing Inc., Cornelius, OR, USA). Fresh fecal slurry (20% *w/v*) was prepared in the phosphate buffer saline (PBS; pH 8.0) and homogenized using a high-speed stomacher for 3 min before inoculation into each fermentation test. A modified anaerobic batch fermentation method from Charoensiddhi, Conlon [21] was used to assess the effect of polyphenols from MSE on gut microbiota composition and SCFA production. Fresh fecal samples were used as inoculum with 1.0% (*w/v*) in each fermentation. Sample substrates (MSE, GA, and VIT at a concentration of 0.1% (*w/v*) GAE in fermentation media were used in each test, and no substrate was added for the blank (CON). The fermentation positive control was supplemented with fructo-oligosaccharide (FOS) (Nutrition SC Company, Thailand) at a concentration of 1.5% (*w/v*) were included. Test substrates and controls were fermented in triplicate in a batch system fermenter. The fermentation medium contained the following: 2.0 g peptone, 2.0 g yeast extract, 2 g NaHCO_3_, 0.5 g bile salt, 0.5 g L-cysteine, 0.1 g NaCl, 0.05 g Hemin, 0.04 g K_2_HPO_4_, 0.04 g KH_2_PO_4_, 0.01 g MgSO_4_.7H_2_0, 0.01 g CaCl_2_.2H_2_0, 10 μL Vitamin K, 2 mL Tween 80 and 998 mL distilled water. Fermentation was conducted at 37 °C and pH 6.65–6.95 using a pH controller adjusted by 0.5 M HCl and 0.5 M NaOH under anaerobic conditions for 24 h.

#### 2.5.2. SCFA, Phenol, and *p*-Cresol Determination

The SCFA analysis was performed via gas chromatography with a flame ionization detector, according to the method of Charoensiddhi, Conlon [21] and McOrist, Abell [22] with slight modifications. A standard SCFA mixture containing acetic, propionic, butyric, isobutyric, valeric, isovaleric, and caproic acids, phenol, and *p*-cresol was used for the calculation, adjusting the quantity of each compound based on that of the internal standard. The SCFA concentrations were calculated in μmoL/mL.

#### 2.5.3. Gut Microbiota Analysis

1. 16s-rRNA next-generation sequencing

The DNA extraction of samples after 24 h fermentation was conducted using a ZymoBIOMICS DNA Miniprep Kit (D4300; Zymo Research, Irvine, CA, USA) according to the manufacturer’s instructions. The quantity and quality of extracted DNA samples were determined by a Nano Drop™ 2000c spectrophotometer (Thermo Fisher Scientific, Waltham, MA, USA) and agarose gel electrophoresis. The gut microbiota was analyzed by Illumina NovaSeq 6000 (Illumina, San Diego, CA, USA). The 16S-rRNA sequences were processed using bioinformatics tools. Pair-end reads were first quality-trimmed using BBDUK [23] (read quality > 15 at 3′), and the primers at the 5′ end were removed using seqtk. The resulting sequences shorter than 150 bp were excluded along with their pair. The remaining high-quality reads were then corrected for sequence error, to identify chimeras and merge paired-ends into Amplicon Sequence Variances (ASVs) in R package DADA2 v.1.6 [24] using default parameters. Taxonomy assignment was carried out with QIIME2′s naïve Bayes classifier v 2021.8 [25] SILVA 99% OTU database v.138 [26] using a 70% cutoff. ASV that could not be identified at a phylum level were excluded from the analysis. Microbiome profile differences in each time point were presented as fold changes. The percentage of relative abundance in treatment and control groups at each time point was used to compute the fold change.

2. Quantitative real-time polymerase chain reaction (qPCR)

Changes in bacterial numbers were determined via qPCR after 24 h fermentation with a series of microbe-specific primer pairs using the LightCycler^®^480 (Roche, Penzberg, Germany) based on the method by Plupjeen and Chawjiraphan [27]. Table S1 lists the primer sequences for detecting *Enterobacteriaceae*, *Bifidobacterium*, *Bacteroides fragilis*, *Lactobacillus*, *Faecalibacterium prausnitzii*, *Prevotella*, *Clostridium leptum*, *Clostridium coccoides*, *Eubacterium rectale*, and “total bacteria”. PCR reaction mixtures (10 μL each) contained 5 μL of SsoAdvanced™ Universal SYBR^®^ Green Supermix (Bio-Rad, Hercules, CA, USA), 3.2 μL of nuclease-free water, 0.4 μL of each forward and reverse primers (to a final concentration of 10 μmoL), and 1 μL of adjusted template DNA (not more than 30 ng/μL). The thermal cycling conditions consisted of one cycle of 95 °C for 5 min (initial denaturation) followed by 45 cycles of denaturation at 95 °C for 10 s, primer annealing for 10 s at the optimal temperatures (shown in Table S1), and an extension step at 72 °C for 10 s. Standard curves were constructed using specific primers to amplify the genomic DNA. Each PCR product was cloned into a pGEM-T Easy vector according to the manufacturer’s instructions (Promega, Madison, WI, USA). The recombinant plasmids were prepared with 10-fold serial dilutions of the 16S-rRNA gene to copy numbers of 10^1^–10^9^. The serial dilution series in each group was used as a template for the standard curve.

### 2.6. Anti-Diabetic Activity

The human hepatocyte carcinoma cell line (HepG2) was purchased from the American Type Culture Collection (ATCC, Rockville, MD, USA). HepG2 cells were cultured according to a previous study [28], grown in DMEM (1 g/L glucose) containing 10% FBS, 1% penicillin and streptomycin, and 1% nonessential amino acids. Cells were maintained at 37 °C in a humidified atmosphere with 5% CO_2_. The medium was changed every other day, and experiments were typically performed with cells at 80–90% confluence. The MSE was diluted with 50% ethanol and filtered through a 0.2 μm membrane filter right before use. The MSE solution was diluted with medium to obtain the desired concentrations.

#### 2.6.1. Establishment of Insulin-Resistant HepG2 Cells

To determine the effects of MSE on intracellular reactive oxygen species (ROS), insulin resistance was induced in HepG2 cells according to a previous study [28]. HepG2 cells were seeded at a density of 4 × 10^4^ cells/well in a 96-well plate. After reaching confluence the next day, the medium was gently removed and replaced with FBS-free DMEM (4.5 g/L glucose) supplemented with 1 μM insulin for 24 h to induce insulin resistance. The HepG2 cells were used for further studies.

The viability of HepG2 cells was determined using the (3-(4,5-dimethylthiazol-2-yl)-2,5-diphenyltetrazolium bromide, a tetrazole) (MTT) assay according to a previous study [28]. Control cells were treated with DMEM, and cells were treated with 1% ethanol as the internal control.

#### 2.6.2. Determination of Intracellular ROS in Insulin-Resistant HepG2 Cells

The intracellular ROS was determined according to a previous study [29]. Briefly, insulin-resistant HepG2 cells were treated with FBS-free DMEM containing MSE at different concentrations (25–400 μg/mL) and incubated for 24 h. Then, 10 μM DCFH-DA was added to each well. After a 30 min incubation, cells were washed with an FBS-free medium to remove excess dye. The fluorescence intensity was measured using a microplate reader (SparkTM 10M multimode, TECAN, Männedorf, Switzerland) at an excitation wavelength of 485 nm with an emission wavelength of 530 nm. The percentage of intracellular ROS was estimated by quantifying fluorescence intensity and calculated using the following equation:

(1) $$\%\;\mathrm{Intracellular}\;\mathrm{ROS}=\frac{\mathrm{Fluorescence}\;\mathrm{intensity}\;\mathrm{of}\;\mathrm{cells}\;\mathrm{treated}\;\mathrm{with}\;\mathrm{MSE}}{\mathrm{Fluorescence}\;\mathrm{intensity}\;\mathrm{of}\;\mathrm{the}\;\mathrm{control}\;\mathrm{cells}}\times 100$$

#### 2.6.3. Determination of Cellular Glucose Uptake in Insulin-Resistant HepG2 Cells

Cellular glucose uptake in insulin-resistant HepG2 cells was determined according to the previous study [28]. Briefly, insulin-resistant HepG2 cells were treated with FBS-free DMEM containing MSE at different concentrations (25–400 μg/mL) and incubated for 24 h. Then, the medium was replaced with 100 nM insulin in phosphate buffer solution (PBS) and the cells were incubated for another 30 min. Thereafter, 10 μL of 2-NBDG were added to obtain a final concentration of 40 μM. The cells were incubated at 37 °C for 1 h. Then, the cells were washed twice with chilled PBS. The fluorescence intensity was immediately measured at an excitation wavelength of 485 nm and an emission wavelength of 528 nm using a microplate reader (SparkTM 10M multimode, TECAN, Männedorf, Switzerland). The percentage of cellular glucose uptake was estimated by quantifying fluorescence intensity and calculated using the following equation:

(2) $$\%\;\mathrm{Cellular}\;\mathrm{glucose}\;\mathrm{uptake}\;=\frac{\mathrm{Fluorescence}\;\mathrm{intensity}\;\mathrm{of}\;\mathrm{cells}\;\mathrm{treated}\;\mathrm{with}\;\mathrm{MSE}}{\mathrm{Fluorescence}\;\mathrm{intensity}\;\mathrm{of}\;\mathrm{the}\;\mathrm{control}\;\mathrm{cells}}\times 100$$

### 2.7. Anti-Inflammatory Activity

Human monocyte THP-1 cells (ATCC; Rockville, MD, USA) were grown in RPMI 1640 medium supplemented with 10% FBS and 1% penicillin–streptomycin in a humidified incubator at 37 °C and 5% CO_2_. Exponential-phase THP-1 monocytes with passage numbers less than 25 were simultaneously stimulated with 100 ng/mL LPS from *Escherichia coli* (O111:B4) and different concentrations of MSE (4, 8, and 16 μg/mL) for 3 h. The MSE concentrations were selected according to cell viability (>90%) via the MTT assay, as described by Chanput, Reitsma [30]. The expression of proinflammatory genes TNF-α, IL-1β, IL-6, and IL-8 was measured using qPCR with primer sequences indicated in Chanput, Mes [31]. Glyceraldehyde-3-phosphate dehydrogenase and the 0 h time point of nonstimulated cells were used for the ΔΔCt normalization. Results were expressed as the relative fold change [31].

### 2.8. Statistical Analysis

All measurements were performed in a triplicate independent analysis for each sample. The results of SCFA, gut microbiota, and bacterial population are expressed as means and standard error of means, otherwise are expressed as means and standard deviations. For only the results of gut microbiota, the analysis of sequencing information was performed regarding the normality of data distribution calculated using the Shapiro–Wilk algorithm. Nonparametric statistical analysis was selected for this analysis. The significance of categorical taxa was determined using the Kruskal–Wallis test and Dunn’s posthoc analysis at a 95% confidence interval [32]. Correction for multiple comparison analysis was performed through Bonferroni adjustments. All statistical analyses and visualizations were carried out in XLSTAT 2019.2.2 and GraphPad Prism 8.4.3. For other results, statistical analyses were performed using IBM SPSS Statistics Version 28.0 (Thaisoftup Co., Ltd., Bangkok, Thailand). One-way analysis of variance was used to compare the means, and differences were considered significant at *p* < 0.05 by Tukey’s test.

## 3. Results

### 3.1. Chemical Determination and Antioxidant Activity of the Extract

The chemical composition of MSE was analyzed (Table 1). MSE consisted of 3.25% moisture, 0.10% fat, 1.93% crude fiber, 6.67% protein, and 7.13% ash. The TPC of MSE was 320.50 ± 25.66 mg GAE/g extract. The ABTS scavenging activity of MSE was 1190.32 ± 42.77 mg TE/g extract. The remaining was carbohydrates (approximately 80.92%).

### 3.2. Gut Microbiota Modulation

This study explored the impact of polyphenols from MSE on SCFA production and gut microbiota compositions via 16S-rRNA sequencing using human fecal batch fermentation. Fermentation with MSE was compared with fermentations with GA, VIT (commercial polyphenol substrates), FOS (a commercial prebiotic), and the control (no added substrates).

### 3.3. Short-Chain Fatty Acid Production

**Figure 1:**
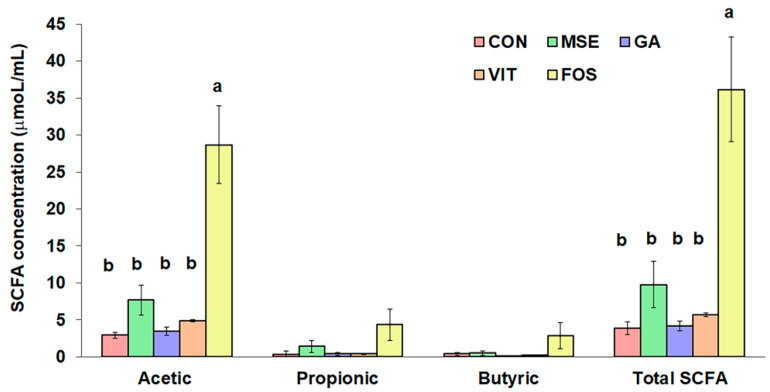
Comparison of short-chain fatty acid (SCFA) concentrations following 24 h of fecal fermentation with mung bean seed coat extract (MSE), gallic acid (GA), vitexin (VIT), fructo-oligosaccharide (FOS), and no added substrate (control; CON). Values are means ± standard error of the mean (SEM) (*n* = 3). Means with different letters in the same SCFA are significantly different (*p* < 0.05).

The results in Figure 1 demonstrate the production of acetic, propionic, and butyric acid in all samples, increasing during the fermentation with the highest concentrations at 24 h (data not shown). Acetic acid was the dominant SCFA produced during fermentation. Although the total SCFA concentrations of FOS-containing treatments (36.2 μmol/mL) were significantly greater (*p* < 0.05) than those of other tested samples, fermentations with MSE, GA, and VIT treatment showed higher total SCFA production than the control (3.9 μmol/mL), especially with MSE (9.8 μmol/mL). Nevertheless, there were no significant differences (*p* > 0.05) in propionic and butyric acid concentrations among the tested samples. The acetic acid showed the same trend as the total SCFA formation. All samples produced low levels of isobutyric, valeric, isovaleric, and caproic acids, as well as phenols and *p*-cresol (data not shown).

### 3.4. Gut Microbiota Compositions

**Figure 2:**
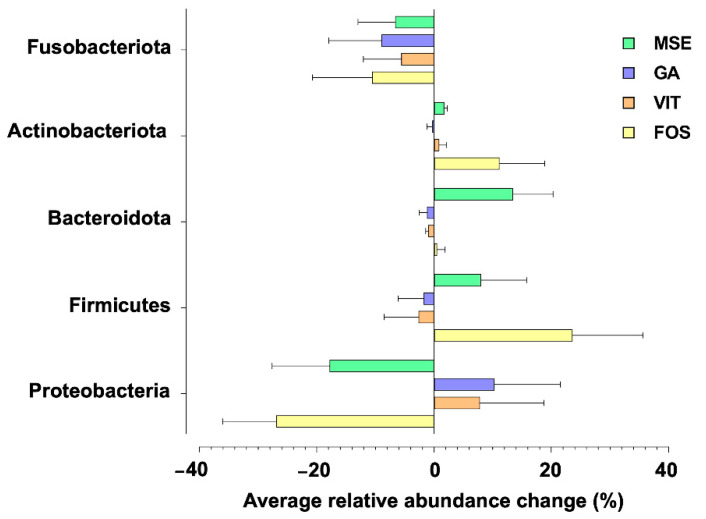
Phylum-level changes in the gut microbiota upon fermentation with mung bean seed coat extract (MSE), gallic acid (GA), vitexin (VIT), and fructo-oligosaccharide (FOS), compared with no added substrate (control; CON) following 24 h fecal fermentations. Only taxa with relative abundances higher than 0.5% were included.

Figure 2 shows the phylum-level taxonomic compositions of tested samples. Relative to the control, a clear decrease in the relative abundance of Proteobacteria and Fusobacteriota, common potentially pathogenic bacteria groups, was observed in the fermentations supplemented with MSE and FOS, whereas a decrease of Fusobacteria was found only in the GA- and VIT-containing treatments. Conversely, an increase in the relative abundance of generally beneficial gut microbiota groups such as Firmicutes, Bacteroidota, and Actinobacteriota was observed in fermentations supplemented with MSE and FOS, compared with their abundance in the fermentations supplemented with GA and VIT. 

**Figure 3:**
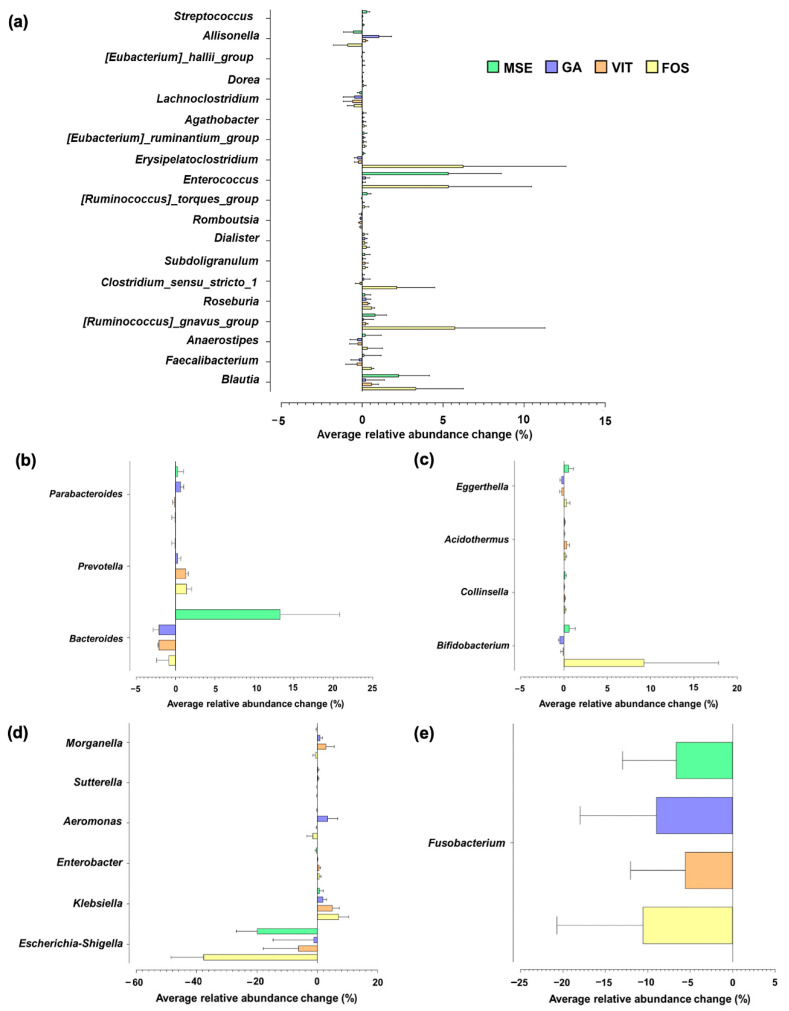
Genus-level changes in the gut microbiota of fermentations with mung bean seed coat extract (MSE), gallic acid (GA), vitexin (VIT), and fructo-oligosaccharide (FOS), compared with the microbiota of fermentation without added substrate (control; CON), following 24 h of fecal fermentation. Only taxa with relative abundances higher than 0.15% were included. Each taxon was grouped based on its phylum level: (**a**) Firmicutes, (**b**) Bacteroidota, (**c**) Actinobacteriota, (**d**) Proteobacteria, and (**e**) Fusobacteriota.

Microbial growth stimulation by MSE was observed in some genus-level classifications, when compared with the growth in the control group. An increase in the abundance of butyrate producers such as *Enterococcus*, *Ruminococcus*, and *Blautia* was observed upon MSE and FOS supplementation (Figure 3a). Furthermore, the proportion of *Bacteroides* was higher upon supplementation with MSE but not with GA, VIT, and FOS (Figure 3b). However, the abundance of *Parabacteroides* and *Prevotella* increased when the GA and VIT were supplemented, respectively. Additionally, an increase in the level of *Bifidobacterium* were observed in fermentations with FOS and MSE (Figure 3c). Apart from the changes in beneficial bacteria, the modulation of potential pathogenic bacteria was observed in Figure 3d and e. Relative to the control, a decrease in the relative abundance of *Escherichia* and *Shigella* was especially found in fermentations with MSE and FOS. In addition, the lowest increase in the relative abundance of *Klebsiella* was observed in fermentations supplemented with MSE compared with other samples.

In addition to 16S-rRNA sequencing, qPCR was performed to confirm the effects of MSE on selected bacteria present in human feces over 24 h of fermentation (Table 2). These target gut microbes were selected on the basis of their relevance to gut health. Relative to that in the control, the number of total bacteria significantly increased (*p* < 0.05) in all other tested samples, particularly in fermentations with MSE and FOS. A significant increase (*p* < 0.05) in the numbers of beneficial bacteria such as *Bifidobacterium*, *Lactobacillus*, *Faecalibacterium prausnitzii*, and *Prevotella* was also observed in MSE-, GA-, VIT-, and FOS-supplemented fermentations with more dominance in the MSE and FOS samples. However, we did not notice the changes in other main butyrate-producing bacteria such as *Clostridium leptum* and *Clostridium coccoides*/*Eubacterium rectale*. There were also no significant differences (*p* > 0.05) in the numbers of *Enterobacteriaceae* and *Bacteroides fragilis*, which are often linked to poor gut health outcomes compared to controls. All results indicated that the MSE-containing treatment showed a greater improvement of beneficial bacteria numbers than the other polyphenol-rich substrates, GA and VIT, but still lower than that of FOS in both 16S-rRNA sequencing and qPCR results.

### 3.5. Intracellular ROS in Insulin-Resistant HepG2

The liver is the major organ for regulating blood glucose. Thus, we used HepG2 cells to investigate the anti-diabetic activity of MSE. We investigated the cytotoxicity of MSE on HepG2 cell survival after 24 h treatment with MSE. Since MSE was dissolved in ethanol, a 1% ethanol treatment was used as a control. The survival of HepG2 cells was reduced by >20% with MSE concentrations more than 400 μg/mL (data not shown). Hence, MSE up to 400 μg/mL was used for further experiments.

**Figure 4:**
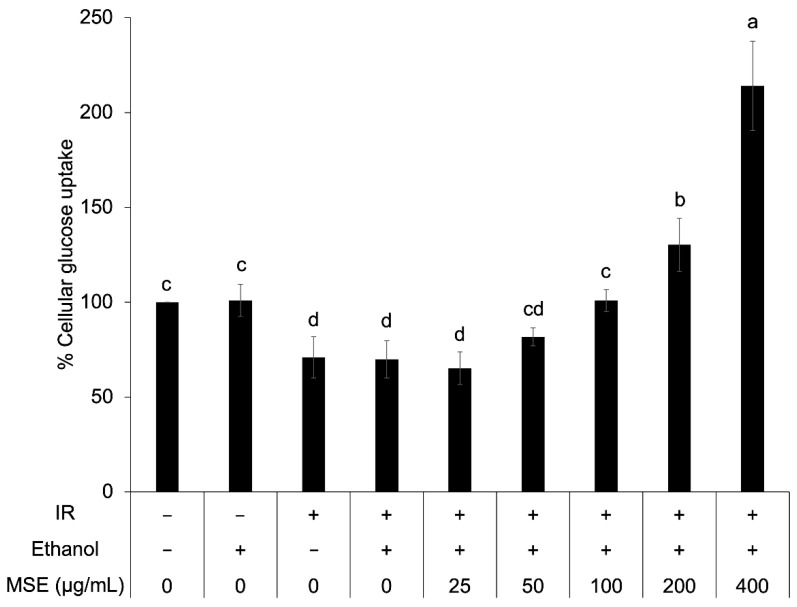
Intracellular reactive oxygen species (ROS) in the control cells and the insulin-resistant cells (IR) with and without mung bean seed coat extract (MSE) treatment. Statistical analyses were performed using Tukey’s posthoc test one-way analysis of variance (ANOVA). Values are expressed as the mean ± SD (*n* = 3). Different letters (**a**–**d**) indicate statistical differences (*p* < 0.05). IR is insulin-resistant HepG2 cells.

The antioxidant activity of MSE was further investigated in insulin resistant HepG2 cells. The percentage of intracellular ROS in insulin-resistant HepG2 cells was significantly higher than that of control cells (Figure 4). The percentage of intracellular ROS decreased with increasing concentrations of MSE. The reduction of intracellular ROS via MSE treatment indicated the reduction of oxidative stress in insulin-resistant HepG2 cells.

### 3.6. Cellular Glucose Uptake in Insulin-Resistant HepG2

**Figure 5:**
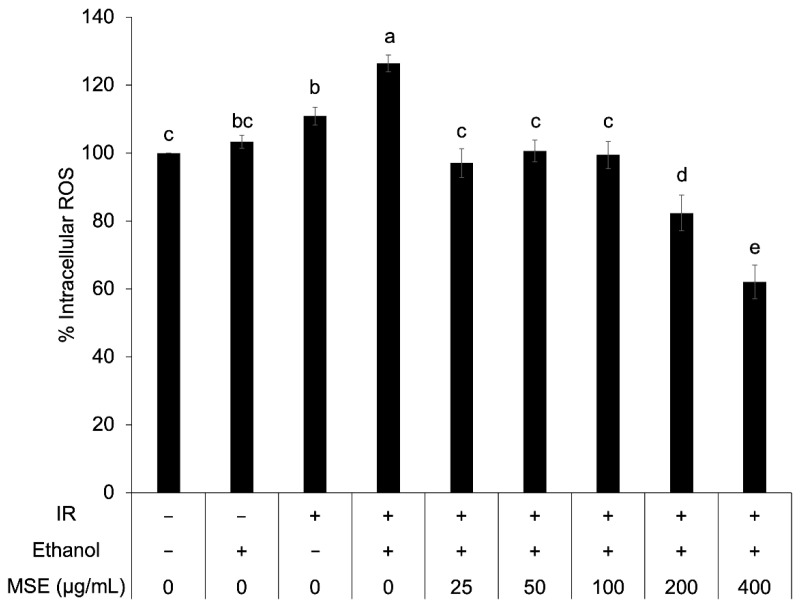
Cellular glucose uptake in the control and insulin-resistant cells with and without mung bean seed coat extract (MSE) treatment. Statistical analyses were performed using Tukey’s posthoc test and one-way analysis of variance (ANOVA). Values are expressed as the mean ± standard deviation, SD (*n* = 3). Different letters (**a**–**e**) indicate statistical differences (*p* < 0.05). IR: insulin-resistant HepG2 cells.

To confirm the anti-diabetic activity of MSE, cellular glucose uptake in insulin-resistant HepG2 cells was determined after insulin stimulation. The insulin-resistant HepG2 cells presented a significantly lower percentage of cellular glucose uptake than that of the control cell (Figure 5). Ethanol did not have an effect on cellular glucose uptake. The result showed that 100–400 μg/mL MSE concentration increased cellular glucose uptake in insulin-resistant HepG2 cells (44.3–206.4%) but not 25–50 μg/mL. This indicated that MSE at a higher dose improved insulin sensitivity of insulin-resistant HepG2 cells.

### 3.7. Anti-Inflammatory Activity

**Figure 6:**
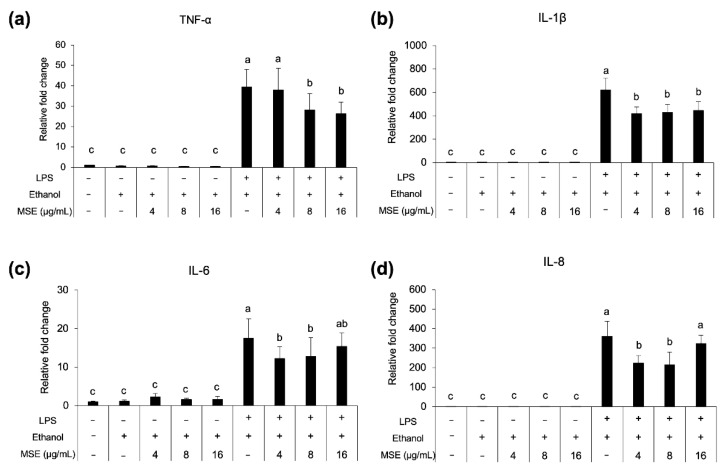
Proinflammatory cytokine gene TNF-α (**a**), IL-1β (**b**), IL-6 (**c**) and IL-8 (**d**), expression of THP-1 monocytes treated with MSE with and without LPS stimulation and nonstimulated cells (control). Statistical analyses were performed using Tukey’s posthoc test and one-way analysis of variance (ANOVA). Values are expressed as the mean ± SD (*n* = 3). Different letters indicate statistical differences (*p* < 0.05).

The LPS concentration in MSE, tested with a LAL Chromogenic Endotoxin Quantification kit, was 20 μg/mL (prepared at 50 mg extract/mL). Cell viability was reduced by >10% with MSE concentrations >120 μg/mL (data not shown). The ethanol control did not affect THP-1 cell viability. According to the LPS concentration >10 pg/mL could up-regulate proinflammatory genes [33], thus, the ceiling of MSE testing concentrations for THP-1 monocytes could not be higher than 24 μg/mL. As shown in Figure 6, MSE at concentrations of 4, 8, and 16 μg/mL reduced the expression of proinflammatory genes TNFα, IL-1β, IL6, and IL8 in LPS-stimulated THP-1 monocytes, in a dose-independent manner. No expression of the aforementioned genes was detected in LPS-unstimulated THP-1 cells. These results implied the anti-inflammatory activity of MSE.

## 4. Discussion

The present study extracted polyphenols from mung bean seed coat via PLE using 50% ethanol as an extracting solvent. The content of phenolic compounds in MSE obtained from PLE (320.50 mg GAE/g extract) was much higher than that in previous studies using hot water for extraction from whole mung bean (60.27 mg GAE/g extract) [28] and mung bean seed coat (4.39 mg GAE/g extract) [9]. When high temperatures and pressures are applied in the extraction process, they can promote the recovery of phenolic and flavonoid compounds from plant substances, as is the case in jabuticaba skins [34], cocoa bean shells [35], and rice grains [36]. High temperatures and pressures, such as the extracting conditions used in this study (160 °C and 1500 psi), can breakdown the plant matrix, thus enhancing the diffusion of the target compounds to the surface and releasing to the solvent [36,37]. The high carbohydrate content of MSE (approximately 80%) may consist of polysaccharides with an arabinogalactan backbone [6] as main components.

SCFA, particularly acetic, propionic, and butyric acid, are important metabolites in gut microbial fermentation. Several recent studies have highlighted their role in immune regulation in innate and adaptive immunity and the prevention of several diseases [38]. In this study, the influence of polysaccharides, which is one of main components in MSE and FOS might have a greater contribution to the production of SCFA than that of polyphenol substrates; GA, and VIT. Several polyphenols were demonstrated to have potential antimicrobial activities [39], which might suppress the growth of some SCFA-producing bacteria, resulting in lower SCFA production. It was noticed that an increase in acetic acid formation in the fermentation supplemented with MSE may relate to the increase of some acetate producers such as *Bacteroides*, *Ruminococcus*, *Blautia*, and *Bifidobacterium* [40]. We also identified the low production of phenols and *p*-cresol in all samples, indicating the limited level of bacterial metabolites from protein fermentation. The accumulation of phenols and *p*-cresol has been linked with gut diseases [41].

For gut microbiome modulation, fermentation with MSE increased the abundance of some butyrate producers compared to controls. Existing research reported the importance of butyric acid for colonic health promotion, resulting in decreased risks of colon cancer and inflammatory bowel disease [42]. Furthermore, the number of *Bacteroides* was higher in MSE-supplemented conditions, and this was not observed in the fermentation with GA, VIT, and FOS. This could be due to the ability of this bacterium to use some specific polysaccharides contained in the MSE. *Bacteroides* possess large amounts of carbohydrate-active enzymes (CAZymes), which can break down plant cell walls [43]. Many studies demonstrated that plant polysaccharides can be fermented by gut microbiota to promote SCFAs production and improve gut microbial community. Therefore, the determination of mung bean polysaccharide structure is important for further investigation in order to understand the structure-activity relationship. An increase in the levels of *Parabacteroides* and *Prevotella* was observed on GA and VIT supplementation. The low abundance of *Parabacteroides* is related to patients with obesity and nonalcoholic fatty liver [44,45], whereas *Prevotella* is in abundance in humans consuming a fiber-rich diet. However, a previous study reported that the consumption of red wine significantly increased the abundance of *Prevotella* [46], as well as the pomegranate extracts [47]. Moreover, long-term fermentation in an *in vitro* SHIME model of sea buckthorn berry juice promoted the growth of *Prevotella* [48]. Additionally, an increase in the number of *Bifidobacterium*, a known marker of prebiosis [40], was observed in fermentations with FOS and MSE, compared with controls. Conversely, the low abundance of potentially pathogenic bacteria such as *Escherichia* and *Shigella* was found in the MSE- and FOS-containing treatments. *Escherichia* and *Shigella* are Gram-negative bacteria, which could induce inflammatory reactions, ulceration, and bloody or mucoidal diarrhea [49]. Additionally, the lowest increase in the relative abundance of *Klebsiella* was observed in fermentations supplemented with MSE. This bacterium appears to be found in patients with gastroenteritis, as opposed to control groups [50]. In this study, qPCR results corresponded to those from 16S-rRNA sequencing. Relative to the control group, fermentation with MSE influenced the growth of beneficial gut microbiota, although there was no clear reduction in potentially pathogenic bacteria determined via qPCR. This might be because of the high carbohydrate component of MSE, moreover its polyphenol enrichment. There is increasing interest in bioactive polysaccharides and polyphenols obtained from many plant materials as prebiotics demonstrating gut health benefits. A previous study reported that the dietary polysaccharides from mung bean skin could improve gut health [16]. This polysaccharide regulated gut microbiota compositions in mice by promoting the growth of Firmicutes and Bacteroidetes and descending the number of TM7. Additionally, pure and rich-polyphenol extracts and dietary sources from soy products, cocoa, fruit, tea, and wine revealed several beneficial effects on human gut health [39].

Imbalances in the production and elimination of ROS lead to oxidative stress, which modulates intracellular signaling, including insulin signaling and inflammatory status [51]. Bioactive compounds with the ability to reduce intracellular ROS levels can usually mitigate oxidative stress and the development of insulin resistance and inflammation. It was shown that MSE increased superoxide dismutase, catalase, and glutathione peroxidase activity in *db/db* mice through its antioxidative activity [11]. Saeting andChandarajoti [28] reported that mung bean water extract increased the percentage of cellular glucose uptake in insulin-resistant HepG2 cells. Ethanolic MSE also reduced fasting blood glucose in *db*/*db* mice and KK-Ay mice [11,12]. The glucose-lowering effect of seven phenolic compounds, including agrimonolide, desmethylagrimonolide, quercetin, luteolin, luteolin-7-*O*-glucoside, kaempferol, and apigenin were also reported [52]. Chanput and Krueyos [53] showed a correlation between antioxidative and anti-inflammatory activities of flavonoids. Thus, the increase in cellular glucose uptake and reduction of proinflammatory gene expression with MSE treatment might be explained by the reduction of intracellular ROS and oxidative stress.

## 5. Conclusions

Polyphenol extract from MSE could increase the levels of SCFA produced by fermentation, compared with those of controls. The analysis of gut microbiota by 16S-rRNA sequencing showed that MSE supplementation not only promoted the relative abundance of beneficial bacteria (*Enterococcus*, *Ruminococcus*, *Blautia*, *Bacteroides Bifidobacterium*, *Lactobacillus*, *Faecalibacterium prausnitzii*, and *Prevotella*) but also decreased that of potentially pathogenic bacteria (*Escherichia-Shigella*). Although polyphenols play a role in gut microbiota modulation, polysaccharides in MSE seemed to have more influence. MSE exerted anti-diabetic activity in insulin resistant HepG2 cells, by reducing intracellular reactive oxygen species and increasing cellular glucose uptake. Moreover, MSE showed anti-inflammatory activities in LPS-stimulated THP-1 monocytes by reducing expression of TNFα, IL-1β, IL-6, and IL-8 genes. These findings indicated that MSE have the potential to be developed as functional food ingredients with gut health benefits, as well as anti-diabetic and anti-inflammatory properties. Taken together, the positive results from our *in vitro* studies highlight the importance of conducting further animal and clinical studies on MSE.

## Figures and Tables

**Table 1:** Chemical composition and antioxidant activity of MSE (wet basis).

Parameter	Composition
Moisture	3.26 ± 0.07%
Fat	0.10 ± 0.01%
Crude fiber	1.93 ± 0.07%
Protein	6.67 ± 0.02%
Ash	7.13 ± 0.01%
Total phenolic content	320.50 ± 25.66 mg GAE/g extract
ABTS scavenging activity	1190.32 ± 42.77 mg TE/g extract

Note: Values are means of three independent experiments (*n* = 3). ABTS: 2,2′-azinobis-(3-ethylbenzothiazoline-6-sulfonic) acid. GAE: gallic acid equivalent. TE: trolox equivalent.

**Table 2:** Bacterial population (log_10_ cell mL^−1^ ± standard error of the mean, SEM) in 24 h batch fermentations with mung bean seed coat extract (MSE), gallic acid (GA), vitexin (VIT), fructo-oligosaccharides (FOS), and no added substrate (control; CON).

Substrates	Total Bacteria	*Bifidobacterium*	*Lactobacillus*	*F. prausnitzii*	*Prevotella*	*C. leptum*	*C.* *coccoides* -*E. rectale*	*Entero* *bacteriaceae*	*B. fragilis*
CON	9.55 ± 0.10 ^c^	7.79 ± 0.07 ^c^	6.47 ± 0.11 ^b^	7.20 ± 0.49 ^c^	8.02 ± 0.18 ^c^	6.72 ± 0.53	8.24 ± 0.52	10.57 ± 0.47	7.51 ± 0.64
MSE	11.48 ± 0.13 ^a^	9.80 ± 0.16 ^b^	9.10 ± 0.26 ^a^	9.20 ± 0.30 ^a^	9.38 ± 0.51 ^b^	8.77 ± 0.21	10.04 ± 0.39	11.84 ± 0.28	9.44 ± 0.94
GA	10.37 ± 0.22 ^b^	9.55 ± 0.83 ^b^	8.51 ± 0.41 ^a^	7.88 ± 0.08 ^b^	8.45 ± 0.14 ^b^	7.20 ± 0.10	8.94 ± 0.34	11.27 ± 0.29	7.83 ± 0.79
VIT	10.43 ± 0.47 ^b^	8.81 ± 0.08 ^b^	8.28 ± 0.25 ^a^	7.91 ± 0.31 ^b^	8.74 ± 0.42 ^b^	7.35 ± 0.34	8.78 ± 0.56	11.39 ± 0.23	7.98 ± 0.61
FOS	11.42 ± 0.30 ^a^	9.91 ± 0.47 ^a^	9.23 ± 0.42 ^a^	9.35 ± 0.60 ^a^	10.31 ± 0.70 ^a^	8.73 ± 0.72	9.99 ± 0.75	10.66 ± 0.20	9.54 ± 0.25

Note: Values are means of three independent experiments (*n* = 3), from which DNA was extracted in duplicate. Different letters in the columns with superscripts means represent significant differences (*p* < 0.05).

## Data Availability

The dataset generated for this research are available on request to the corresponding author.

## Supplementary Materials

The following supporting information can be downloaded at: https://www.mdpi.com/article/10.3390/nu14112275/s1, Table S1: Primer sequence, annealing temperature, and size of PCR products [54,55,56,57,58,59,60].
