# Supplementary material for: Gut Microbiota Modulation, Anti-Diabetic and Anti-Inflammatory Properties of Polyphenol Extract from Mung Bean Seed Coat (Vigna radiata L.)
Source: Nutrients. 2022 May 28;14(11):2275. doi: 10.3390/nu14112275 (PMC9182904; doi:10.3390/nu14112275)
Supplement: Supplementary file 1 [file nutrients-14-02275-s001.zip › nutrients-1746653-supplementary.pdf]

Table S1 Primer sequence, annealing temperature, and size of PCR products

Target	Primer sequence	Tm (ºC)	Size (bp)	Reference
Total bacteria	ACTCCTACGGGAGGCAGCAGT<br>GTATTACCGCGGCTGCTGGCAC	56	200	Tannock, Munro, Harmsen, Welling, Smart and Gopal [1]
Bifidobacterium spp.	TCGCGTC)C/T(GGTGTGAAAG<br>CCACATCCAGC)A/G(TCCAC	58	243	Rinttilä, Kassinen, Malinen, Krogius and Palva [2]
Bacteroides fragilis group	ATAGCCTTTCGAAAGRAAGAT<br>CCAGTATCAACTGCAATTTTA	50	501	Matsuki, Watanabe, Fujimoto, Miyamoto, Takada, Matsumoto, Oyaizu and Tanaka [3]
Enterobacteriaceae	CATTGACGTTACCCGCAGAAGAA GC<br>CTCTACGAGACTCAAGCTTGC	57	195	Bartosch, Fite, Macfarlane and McMurdo [4]
Clostridium coccoides – Eubacteria. rectale group	CGGTACCTGACTAAGAAGC<br>AGTTT(C/T)ATTCTTGCGAACG	51	429	Rinttilä, Kassinen, Malinen, Krogius and Palva [2]
Clostridium leptum group	GCACAAGCAGTGGAGT<br>CTTCCTCCGTTTTGTCAA	55	239	Matsuki, Watanabe,

				Fujimoto, Takada and Tanaka [5]
F. prausnitzii-<br>*S. variabile* group	ACCATGAGAGCCGGGGGG<br>GGTTACCTTGTTACGACTT	57	100	Lund, Bjerrum and Pedersen [6]
Lactobacillus group	AGCAGTAGGGAATCTTCCA<br>CACCGCTACACATGGAG	53	341	Walter, Hertel, Tannock, Lis, Munro and Hammes [7]
Prevotella	CACRGTAAACGATGGATGCC<br>GGTCGGGTTGCAGACC	62	513	Matsuki, Watanabe, Fujimoto, Miyamoto, Takada, Matsumoto, Oyaizu and Tanaka [3]

References for Table S1

[1] G. W. Tannock, K. Munro, H. J. M. Harmsen, G. W. Welling, J. Smart, P. K. Gopal, *Applied and Environmental Microbiology*. **2000**, *66*, 2578-2588.

[2] T. Rinttilä, A. Kassinen, E. Malinen, L. Krogius, A. Palva, *Journal of Applied Microbiology*. **2004**, *97*, 1166-1177.

[3] T. Matsuki, K. Watanabe, J. Fujimoto, Y. Miyamoto, T. Takada, K. Matsumoto, H. Oyaizu, R. Tanaka, *Applied and Environmental Microbiology*. **2002**, *68*, 5445-5451.

[4] S. Bartosch, A. Fite, G. T. Macfarlane, M. E. T. McMurdo, *Applied and Environmental Microbiology*. **2004**, *70*, 3575-3581.

[5] T. Matsuki, K. Watanabe, J. Fujimoto, T. Takada, R. Tanaka, *Applied and Environmental Microbiology*. **2004**, *70*, 7220-7228.

[6] M. Lund, L. Bjerrum, K. Pedersen, *Poultry Science.* **2010**, *89*, 1217-1224.

[7] J. Walter, C. Hertel, G. W. Tannock, C. M. Lis, K. Munro, W. P. Hammes, *Applied and Environmental Microbiology*. **2001**, *67*, 2578-2585.
